# Investigating the ability to read others’ intentions using humanoid robots

**DOI:** 10.3389/fpsyg.2015.01362

**Published:** 2015-09-09

**Authors:** Alessandra Sciutti, Caterina Ansuini, Cristina Becchio, Giulio Sandini

**Affiliations:** ^1^Departments of Robotics, Brain and Cognitive Sciences, Istituto Italiano di TecnologiaGenoa, Italy; ^2^Department of Psychology, Centre for Cognitive Science, University of TorinoTorino, Italy

**Keywords:** motor cognition, second-person interaction, contingency, kinematics, intention reading, human–robot interaction

## Abstract

The ability to interact with other people hinges crucially on the possibility to anticipate how their actions would unfold. Recent evidence suggests that a similar skill may be grounded on the fact that we perform an action differently if different intentions lead it. Human observers can detect these differences and use them to predict the purpose leading the action. Although intention reading from movement observation is receiving a growing interest in research, the currently applied experimental paradigms have important limitations. Here, we describe a new approach to study intention understanding that takes advantage of robots, and especially of humanoid robots. We posit that this choice may overcome the drawbacks of previous methods, by guaranteeing the ideal trade-off between controllability and naturalness of the interactive scenario. Robots indeed can establish an interaction in a controlled manner, while sharing the same action space and exhibiting contingent behaviors. To conclude, we discuss the advantages of this research strategy and the aspects to be taken in consideration when attempting to define which human (and robot) motion features allow for intention reading during social interactive tasks.

## Reading Intentions from Others’ Movement

The ability to attend prospectively to others’ actions is crucial to social life. Our everyday, common-sense capability to predict another person’s behavior hinges crucially on judgments about that person’s intentions, whether they act purposefully (with intent) or not, as well as judgments about the specific content of the intentions guiding others’ actions – what they intend in undertaking a given action (Baldwin and Baird, 2001).

**FIGURE 1:**
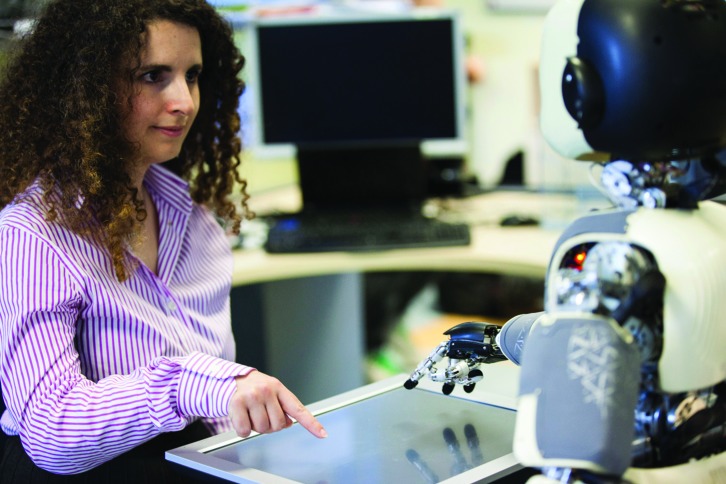
**An illustrative picture of human–robot interaction with the humanoid robot iCub.** The mutual and spontaneous information exchange is mediated by context (i.e., the game on the touch screen that the two partners are playing) and by the agents’ gazing behavior, but also by the intention information embedded in their movement properties. Copyright photo: Agnese Abrusci, Istituto Italiano di Tecnologia© IIT.

Humans rely on several sources to understand others’ intention (**Figure 1**). For instance, by looking at the context of the surrounding environment we are often able to infer what is another person’s intention. If a closed bottle of wine is on the table and a person reaches for a drawer, we guess that he is more probably looking for a bottle opener than for a fork. Under similar circumstances, the information provided by the context would allow an observer to constraint the number of possible inferences, thus facilitating the action prediction process (Kilner, 2011). But actions can also take place in contexts that do not provide sufficient information to anticipate others’ intention. In such cases it has been demonstrated that others’ gaze behavior may be a suitable cue to anticipate the intention to act (Castiello, 2003) as well as the specific goal of an action (Ambrosini et al., 2015). Moreover, there is a growing body of evidence indicating that, in absence of gaze or contextual information, intentions can be inferred from body motion. But how is this possible?

How another agent moves can represent a cue to infer his intention because the way he moves is intrinsically related to his intention. In keeping with previous evidence (e.g., Marteniuk et al., 1987), recent studies have shown that in humans different motor intentions translate into different kinematics patterns (Ansuini et al., 2006, 2008; Sartori et al., 2011b). For instance, Ansuini et al. (2008) asked participants to reach for and grasp the very same object (i.e., a bottle) to accomplish one of four possible actions (i.e., pouring, displacing, throwing, or passing). Kinematic assessment revealed that when the bottle was grasped with the intent to pour, the fingers were shaped differently than in the other conditions. Further studies have extended these effects to the domain of social intention, reporting that not only the presence of a social vs. individual intention (Becchio et al., 2008b), but also the type of “social” intention (compete vs. cooperate) has an effect on action kinematics (Becchio et al., 2008a; see also Georgiou et al., 2007).

Recent evidence suggests that observers are sensitive to early differences in visual kinematics and can use them to discriminate between movements performed with different intentions (Vingerhoets et al., 2010; Manera et al., 2011; Sartori et al., 2011a; Stapel et al., 2012). For instance, Sartori et al. (2011a) tested whether observers use pre-contact kinematic information to anticipate the intention in grasping an object. To this end, they first analyzed the kinematics of reach-to-grasp movements performed with different intents: cooperate, compete against an opponent, or perform an individual action at slow or fast speed. Next, they presented participants with videos representative of each type of intention, in which neither the part of the movement after the grasping, nor the interacting partner, when present, were visible. The results revealed that observers were able to judge the agent’s intent by simply observing the initial reach-to-grasp phase of the action.

The above findings suggest that intentions influence action planning so that different kinematic features are selected depending on the overarching intention. The observer is sensitive to this information and can use it to anticipate the unfolding of an action. Reading intention by observing movement therefore enables humans to anticipate others’ actions, even when other sources of information are absent or ambiguous.

Research on the topic of understanding intention from movement has been traditionally the domain of psychology and neuroscience. However, there is growing interest in applying these ideas to computer vision, robotics, and human–robot interaction (e.g., Strabala et al., 2012; Shomin et al., 2014; Dragan et al., 2015). Unfortunately, the methodologies and paradigms currently used present important limitations. In the next sections, we will first briefly describe the methods traditionally applied to investigate this topic, and we will point out their potential shortcomings. Thereafter we will propose a new potential role for robots: before becoming anticipatory companions, robots could serve as suitable tools to overcome these limitations in research.

## Barriers to Investigation of Intention-from-Movement Understanding

Reading intention from movement observation has been traditionally investigated with *video clips* used as stimuli. In these paradigms, for instance, temporally occluded goal-oriented actions are shown and the participant is asked to watch them and guess which is the actor’s intention. This approach guarantees full control on the stimulation in all its aspects: timing, information content, and perfect repeatability. Moreover, with video manipulation it is also possible to create behaviors that are impossible or unnatural, by modifying selectively relevant properties of the action. However, when looking at a video presentation, the subject is merely an observer, rather than a participant in the interaction. In other words, the use of videos eliminates some fundamental aspects of real collaborative scenarios, including the shared space of actions, the physical presence, the possibility to interact with the same objects and even potential physical contact between the two partners. Furthermore the video paradigm progresses in a fixed design and does not react to the action of the participant. It therefore precludes the possibility to build a realistic interactive exchange. Hence, the use of movie stimuli provides a fundamental way to investigate how others’ actions and intentions are processed, but it should be used to complement other approaches that allow for actual interaction and contingent behavior.

More recently, the use of *virtual reality* systems has been proposed as a tentative solution to this problem. With this kind of settings it is possible to create virtual characters, or avatars, that respond contingently to participant’s behavior (e.g., his gaze or his actions), while still maintaining the proper controllability of video stimuli. This type of methodology has strong potential, but it also has the limitation of detaching the participant from the real world. The resulting subject’s behavior might then be affected not only by actions of the avatar, but also by being immersed in an environment that is not his everyday reality and which might not feature the same physical laws (e.g., gravity). Many aspects of our movements may derive from an optimization or a minimization of energy expenditure computed over life-long interaction with environmental constraints (e.g., Berret et al., 2008). Thus, removing the real environment from the equation could actually cause important changes in the performance of even simple interactions such as passing an object back and forth.

To summarize, the use of video stimuli allows full controllability, but it lacks of the possibility of contingent reaction and compromises the investigation of reading intention-from-motion in the context of a real interaction. On the other hand, virtual reality provides a certain degree of action contingency, but forces the participant to be immersed in a reality, that is different from his everyday experience. Thus, a new tool that goes beyond these limitations and allows an actual interaction with a high level of control is needed. In our opinion, the application of robots may meet these requirements. In the following, we propose a brief description of the main properties that would make robots, especially humanoids, a valuable instrument to investigate human ability to read intentions from others’ movements.

## Humanoid Robots as New Tool to Investigate Intention Understanding

### Second-Person Interaction

As mentioned above, current paradigms investigating intention understanding are often based on a “spectator” approach to the phenomenon. However, social cognition differs in three important ways when we actively interact with others (‘second-person’ social cognition) compared to when we merely observe them (‘third-person’ social cognition; Schilbach and Timmermans, 2013). First, being involved in an interaction has an emotional component that is missing in a detached action observation setting (Schilbach and Timmermans, 2013). Second, it changes the perception of the environment, which is processed in terms of the range of possible actions of the two partners rather than those of the single participant (e.g., Richardson et al., 2007; Doerrfeld et al., 2012). Third, it is characterized by a higher flexibility, as the partners can adaptively change their actions during the interaction itself (e.g., Sartori et al., 2009). Robots provide the unique opportunity to investigate second-person social cognition, by engaging the participant in a face-to-face interaction without losing the controllability of the experiment or the shared environment. Although an experimenter or a human actor can be used as co-agent in a real interaction, the very fact that two people interacting influence each other in a complex way would easily result in behaviors that go beyond experimental control (see Streuber et al., 2011). Moreover, the automatic processes that constitute a great part of implicit communication (e.g., unintentional movements or gazing) are very difficult to restrain. As suggested by Bohil et al. (2011), “an enduring tension exists between ecological validity and experimental control” in psychological research. A robotic platform might provide a way out of this dilemma because it could sense the ongoing events and elaborate the incoming signals through its onboard sensors so to be able to react contingently to the behavior of the human partner, according to predefined rules.

### Modularity of the Control

A further advantage of the use of robotic platforms relates to the possibility to isolate the contributions of specific cues that inform intention-from-movement understanding. When we observe other’s actions, the incoming flow of sensory information provides multiple sources of evidence about the agent’s goal, such as their gaze direction, arm trajectory, and hand pre-shape. The contribution of these factors in isolation is indicated by several empirical studies (e.g., Rotman et al., 2006; Manera et al., 2011). However, how these factors contribute together to mediate intention understanding remains unclear (Stapel et al., 2012; Furlanetto et al., 2013; Ambrosini et al., 2015). It is difficult in practice to separate and independently manipulate individual cues. For instance, the temporal dynamics of eye-hand coordination in a passing action or the relationship between the speed of a reaching movement and its accuracy are not independently planned by a human actor (see Ambrosini et al., 2015). Conversely, on a robot these aspects can be separated, distorted, or delayed, to assess the relative importance of each feature of the motion. For instance, we know that the unfolding of an action kinematics occurs within a specific temporal structure, e.g., the peak deceleration occurs at around 70–80% of a reach-to-grasp movement (Jeannerod, 1986). The robot allows the experimenter to selectively manipulate the time of peak deceleration to assess precisely which temporal deviations from human-like behavior could be tolerated by an observer, without hindering the possibility to infer other’s intentions.

### Shared Environment

Robots are embodied agents, moving in our physical world, and therefore sharing the same physical space, and being subject to the same physical laws that influence our behavior. In contrast to virtual reality avatars, robots bring the controllability and contingency of the interaction into the real-world, where actual interaction usually occurs. Furthermore, robots with a humanoid shape have the advantage of being able to use the tools and objects that belong to a human environment and have been designed for human use. These properties make robots more adaptable to our common environments. In addition, the human shape and the way humans move are encoded by the brain differently with respect to any other kind of shape and motion (Rizzolatti and Sinigaglia, 2010). Consequently, humanoid platforms can probe some of the internal models naturally developed to interact with humans and allow studying exactly those basic mechanisms that make human–human interaction so efficient.

## Necessary Robot Features to Investigate Human Ability to Read Intentions

When using a robot to investigate intention understanding in humans, some potential issues have to be considered. It could be objected, for instance, that the ability to anticipate others’ intentions is strongly related to the properties of the human motor repertoire (Rizzolatti and Sinigaglia, 2010) and a robot does not exactly replicate the shape or the movements of human agent.

Although some researchers have succeeded in copying human appearance quite precisely (Nishio et al., 2007), human movement is indeed much harder to reproduce. This is due, for instance, to the materials and actuators with which robots are built, which are quite dissimilar from human elastic tissues and muscles, and to the complexity of human articulations. Still, entire research areas are devoted to build new robots that more closely resemble motor control and actuation of a human body (e.g., Kenshiro robot, Kozuki et al., 2013).

**FIGURE 2:**
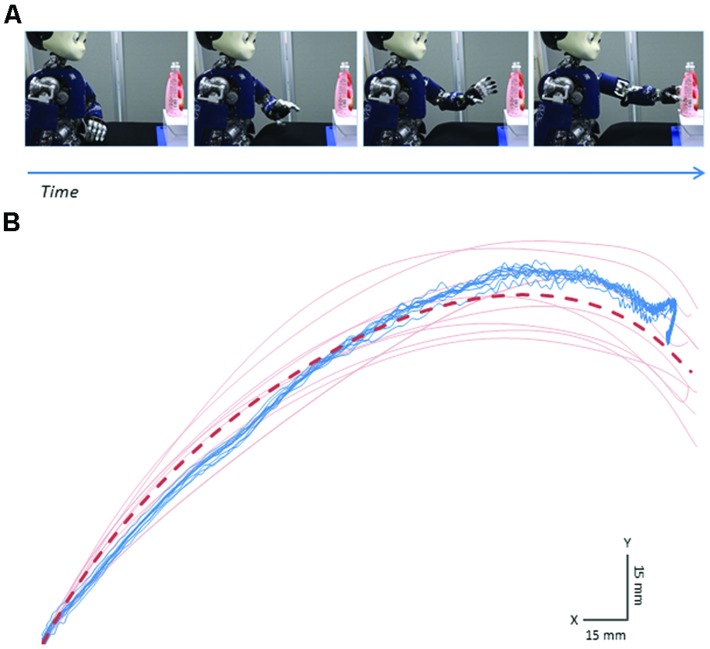
**It is possible to replicate simple movements with a humanoid robot that are *sufficiently* similar to those of a human actor.** Here we show an example where the robot approximates a previously recorded human reaching. **(A)** Snapshots of the humanoid robot iCub reaching for a bottle with the aim to pour its content (i.e., pouring intention). **(B)** Sample trajectories of the palm of the hand on the horizontal (X) and vertical (Y) planes of the motion. Blue lines represent robot actions while red lines indicate human motions. Each line refers to a single movement. Data from ten trials are reported. It can be noticed that robot motion is highly repeatable and reflects quite accurately the average trajectory of the human action to be reproduced (dashed red line). Image by Oskar Palinko.

It is worth noticing that robotic platforms currently available offer interactive contexts in which robotic motion could be *sufficiently* similar to human motion. In this respect, investigation of reading intention-from-movement is particularly suitable for the use of humanoid robots, because it is traditionally focused on simple actions such as reaching to pass, grasping, transporting, or handing-over an object. This choice derives from the observation that most everyday collaborative behaviors are made of combinations of these simple acts. With this “vocabulary” as the focus of interest, it is possible to find existing robotic platforms that allow for human-like visuo-manual coordination, i.e., a control of gaze and manual actions that resembles that of a human (e.g., iCub, Metta et al., 2010, see **Figure 2**). Additionally, an approximate human-like shape, at least in the apparent humanoid structure of the robot body (e.g., torso, arm, hand, neck, head), might be required. This way humans can easily match their own bodily configuration with that of the robot and it is also simpler for experimenters to design robot behaviors approximating human motions both in end-effector and joint trajectories.

Since a robot is not an exact replica of a human, the doubts remain about whether a humanoid actually elicits in the human observers the same class of phenomena that are activated when they are observing a fellow human. A general answer to this question is not available yet (see Sciutti et al., 2012 for a review on the topic). However, there is some evidence suggesting that a humanoid robot exhibiting properly programmed motions can evoke the same automatic behavioral reactions as a human – at least in the context of the simple motions listed before.

One of these phenomena is the automatic anticipation of the action goal of another agent. Such prediction is associated to the activation of the observer’s motor system (Elsner et al., 2013) and therefore does not occur when an object is self-propelling toward a goal position with the same predictable motion (Flanagan and Johansson, 2003). In an action observation task in which the humanoid robot iCub transported an object into a container, the observers exhibited a similar degree of automatic anticipation as for a human actor, suggesting that a comparable motor matching (and goal reading) occurred for both agents (Sciutti et al., 2013a). This result was replicated with another behavioral effect related to motor matching, namely automatic imitation (Bisio et al., 2014). When witnessing someone else performing an action, humans spontaneously adapt their speed to that of their partner. It has been demonstrated that a similar unconscious adaptation occurs also after the observation of a humanoid robot action, but only if robot motion complies with the regularities of human biological motion. Additionally, humans process humanoid and human lifting actions in a similar manner. In line with this, it has been shown that observers are able to infer the weight of an unknown lifted object with the same accuracy both when looking at a human actor or at the iCub robot performing the lifting (Sciutti et al., 2013b, 2014). These results expand previous studies that showed that other behavioral phenomena associated to motor resonance (i.e., the activation of the observer’s motor system during action perception) can generalize to humanoid robot observation, such as priming (Liepelt et al., 2010) and motor interference (Oztop et al., 2005).

Taken together, this evidence indicates that, as far as simple collaborative behaviors are concerned, humanoid robot actions are processed similarly to human actions and trigger a similar response in the human partners. Hence, using a humanoid robot as stimulus could give us insights not only about which mechanisms could facilitate human–robot interaction, but also about the laws subtending the dynamics of human–human interaction.

## Conclusion

We predict that the use of robots as tools for investigating the phenomenon of reading intentions from movement observation will have a substantial impact not only on cognitive science research, but also from a technological standpoint. The tangible benefits for psychology and cognitive science of using humanoid robots to investigate intention reading consist in adding to the research the controllability of each single aspect of interaction (*modularity of control*), a property which is well beyond the possibilities of a human actor, while at the same time preserving a real reciprocity and involvement (*second-person interaction*), also in terms of space (*shared environment*). In turn, the possibility to have robots that move so as to seamlessly reveal their intents, would result in a more efficient, safe, and fluent human-robot collaboration. Indeed, by exploiting the same subtle kinematics signals that enable the timely and rich mutual understanding observed among humans, the implicit reading of robot intentions would happen naturally, with no need of specific training or instructions. Hence this line of research will allow us to build better, more interpretable robots and at the same time to deepen our understanding of the complex field of human–human interaction.

## Conflict of Interest Statement

The authors declare that the research was conducted in the absence of any commercial or financial relationships that could be construed as a potential conflict of interest.
